# Lower Limb Strength Profile in Elderly with Different Pathologies: Comparisons with Healthy Subjects

**DOI:** 10.3390/geriatrics5040083

**Published:** 2020-10-22

**Authors:** Valentina Bullo, Enrico Roma, Stefano Gobbo, Federica Duregon, Manuele Bergamo, Gioia Bianchini, Eleonora Doria, Lucia Cugusi, Andrea di Blasio, Danilo Sales Bocalini, Andrea Ermolao, Marco Bergamin

**Affiliations:** 1 Sport and Exercise Medicine Division, Department of Medicine, University of Padova, Via Giustiniani, 2-35128 Padova, Italy; valentina.bullo@unipd.it (V.B.); romaenrico94@gmail.com (E.R.); federica.duregon@unipd.it (F.D.); manuele.bergamo@gymhub.it (M.B.); andrea.ermolao@unipd.it (A.E.); marco.bergamin@unipd.it (M.B.); 2 GymHub S.r.l., Spin-off of the University of Padova, Via O. Galante 67/a, 35129 Padova, Italy; gioia.bianchini@gymhub.it (G.B.); eleonora.doria@gymhub.it (E.D.); 3 Department of Biomedical Sciences, University of Sassari, 07100 Sassari, Italy; lucia.cugusi@uniss.it; 4 Department of Medicine and Sciences of Aging, G. d’Annunzio University of Chieti-Pescara, Via dei Vestini, 31-66100 Chieti, Italy; andiblasio@gmail.com; 5 Laboratorio de Fisiologia e Bioquimica Experimental, Centro de Educacao Fisica e Deportos, Universidade Federal do Espirito Santo (UFES), Vitoria, ES, Rua Vergueiro, 235, Liberdade, Sao Paulo, SP 01504-00, Brazil; bocaliniht@hotmail.com

**Keywords:** ageing, muscular strength, kidney transplantation, old obese, liver transplantation

## Abstract

Sarcopenia and muscle strength reduction are a frequent disorder in non-communicable chronic diseases. The aims of this study are: (a) to verify if the absolute and relative to body weight muscle strength of lower limb is affected by the presence of pathology; (b) to verify if the trends are different among knee and ankles joints. One-hundred and forty-five elderly were recruited (16 liver transplant recipients, 48 kidney transplant recipients, 52 elderly with obesity, 30 healthy elderly). Muscular strength of lower limb was evaluated. Evaluation protocol included maximal isometric knee extension, maximal isokinetic knee extension and flexion, maximal isokinetic ankle (both right and left) extension and flexion. A statistically significant interaction between measurement and group membership was found for absolute strength measure (F (4.23, 170.56) = 3.316, *p* = 0.011, partial η2 (η2p) = 0.076), and relative strength measure(F (4.44, 174.72) = 16.407, *p* < 0.01, partial η2 (η2p) = 0.294). Elderly patients living with kidney transplants showed the lower level of absolute muscular strength, while relative muscular strength is mainly lacking in the elderly with obesity. The strength profile of elderly subjects is affected by obesity, liver transplantation, and kidney transplantation.

## 1. Introduction

Ageing alone entails a normal decline of physical efficiency as well as the overall physical fitness and body composition. These changes involve muscular strength [1] and peak power output [2], which appears to be related to the loss of muscle mass due to an age-related factor as well as neuromuscular changes [3]. Dynapenia is the reduction of muscle strength without neurological or muscular disease [4]. The degree of muscle loss is variable and depends on different factors. The nervous system’s deterioration affects the control of voluntary skeletal muscle activation, with the reduction of motor units. The loss of muscle strength and power are the consequence of a lower ability of the nervous system to stimulate muscle contraction [5]. Malnutrition, primarily low protein intake, implicates a negative protein balance with the consequences of skeletal muscle atrophy, impaired muscle growth, and functional decline [6]. Physical inactivity is associated with body composition modifications resulting in muscle mass reduction and fat mass increase [7], with consequential reductions of muscular strength. This phenomenon can seriously compromise the wellbeing and the quality of life in the elderly. Indeed, in adults above the age of 60 years, a meta-analysis consisting of 16 prospective and retrospective studies indicated that lower-extremity muscle weakness (OR = 4.9), balance (OR = 3.2), and gait deficits (OR = 3.0) are associated with an increased fall risk [8]. Moreover, age appears to be an important factor that may have an impact on associations between balance and lower-extremity muscle strength/power [9].

Muscle strength is lost more rapidly than muscle mass [10], due to the progressive modification of “muscle quality” with a decrease of fiber number and size [11], micro- and macro-infiltration of fat [11], and impaired neurological modulation of contraction [12]. Generally, lower limbs tend to lose a greater level of muscular strength torque and power than upper limb muscle, probably due to a reduction of physical activity such as walking or running [13,14]. Considering that lower limb muscular strength is necessary to perform daily living activities, the reduction could compromise the independent living maintenance [15].

Muscle strength reduction and sarcopenia are a frequent disorder also in chronic diseases, such as kidney and liver disease, and obesity [16]. The potential mechanisms that may negatively impact skeletal muscle is complex and results from a catabolic state [17] mediated by metabolic acidosis, corticosteroids, and pro-inflammatory stimulus [16], which further promote inflammation. Moreover, physical inactivity is frequent in chronic patients due to a greater decrement in exercise tolerance [17], and different complications, such as cardio-pulmonary changes, hypertension [18], ascites or edema [18], and anemia [17], blocking the practice of regular physical exercise. Hospital recovery for elderly patients with non-communicable diseases is very common. After transplantation, if there are no complications, recovery is not so long, but, before the surgery, dialytic treatments or end-stage liver disease (ESLD) might lead to fatigue [17] or bed rest for long periods [19], resulting in muscle weakness, unsteady gait, and poor balance [20]. Similarly, in patients with obesity, the excess of food intake and scarce physical activity, the low-grade inflammation, insulin resistance and hormonal status may favor the reduction of muscle mass, muscle strength, and weakness [21], with consequent reduction in functional capacity and quality of life [22].

Hence, non-communicable diseases may affect the lower limb strength profile of pathological older adults more than their healthy counterparts, and this could influence the exercise prescription and the adaptation of physical exercise for chronic patients. Therefore, in light of these considerations, the aims of this study are: (a) verify if the absolute and relative to body weight muscle strength of lower limb is affected by the presence of pathology; (b) verify if the trends are different among knee and ankles joints. The research hypothesis is that healthy elderly show relatively higher lower limb muscular strength (adjusted by body weight) than pathological elderly, while we expected the highest absolute muscular strength in elderly with obesity.

## 2. Materials and Methods

### 2.1. Participants

The present work is a secondary data analysis from a clinical database provided by *** Blinded for reviewers***. To obtain a control group, the author used the data from their previous work [23]. The aforementioned clinical database is a collection of data gathered by *** Blinded for reviewers*** with the aim to develop exercise prescription. To match variables between the two databases, only lower limb strength measures were considered. Subjects were divided into 4 sub-groups: “Elderly with obesity group” (OB), “Kidney transplant recipients group” (KTR), “Liver transplant recipients group” (LTR), and “Healthy elderly group” (HEG). The communal inclusion criteria for the 4 sub-groups were ≥60 years old. Specific inclusion criteria were: (a) kidney transplant for the KTR group; (b) liver transplant for the LTR; and (c) Body Mass Index ≥ 30 for OB. Participants were excluded from the investigation if they had a history of cardiovascular, pulmonary, neurologic, musculoskeletal, or other major systemic problems that can negatively influence study results. Moreover, all older adults had no previous experience with isometric or isokinetic muscular test. Each participant was informed about the evaluation purpose procedures, and gave written consent for the treatment of their evaluation results for research purposes accordingly with the Declaration of Helsinki and the following specific guidelines for researchers operating in the interdisciplinary field of exercise and sports sciences [24]. In addition, participants were administered the Mini-Mental State Examination (MMSE) [25], which was used as a screening device to rule out significant cognitive impairments [26]. All procedures were approved by the Ethics Committee of the University of *** (blind for review).

### 2.2. Procedure

Participants’ height and weight were measured with a stadiometer (Ayrton Corporation, Model S100, Prior Lake, MN, USA), an electronic scale (Home Health Care Digital Scale, Model GS 51 XXL, Beuer Gmbh, Ulm, Germany). Height and weight were used to calculate body mass index (BMI) of the participants.

For pathological subjects, medical history, medical examination, and Cardiopulmonary exercise test was administered by a Physician with Sport Medicine specialization (Jaeger-Masterscreen-CPX, Carefusion, Germany). Before muscular strength tests, a warm up was performed to reduce the risk of injuries. A 60-s recovery period was allowed between all testing procedures. Subjects were seated on the multi-joint evaluation system with the backrest angled at 90° to the seat. Belts were placed across the thighs, the pelvis, and the shoulders to minimize body movements and to optimally isolate the movement of knee joints and ankles. Subjects folded their arms across their chest and were not permitted to hold on to the equipment during the tests. Evaluation protocol was previously validated for elderly subjects [23]. The assessed parameters were: maximal isometric bilateral knee extension at 75° of extension, maximal isokinetic bilateral knee extension and flexion with a range of movement between 0° (anatomic 0°) to 85° of knee flexion, right and left maximal isometric ankle plantar, and dorsal flexor at 30° of plantar flexion and right and left maximal isokinetic ankle plantar and dorsal flexor with a range of movement between 0° (anatomic 0°) to 65° of ankle plantar flexion.

During knee trials, the lever fulcrum was aligned with the rotation axis of knee, with the lateral femoral epicondyle used as a landmark, and the shin pad was placed 2 cm above the medial malleoli. Instead, during the ankle trials, the lever fulcrum was aligned with the medial malleoli. Before all isokinetic tests, the weight of the legs and the ankles were noted and a gravity adjustment was made using the computer software.

Four measures were quantified: maximal isometric bilateral knee extension, maximal isokinetic bilateral knee extension and flexion, maximal isometric ankle plantar and dorsal flexion (right and left ankles), and maximal isokinetic ankle plantar and dorsal flexion (right and left ankles). During the maximal isometric bilateral knee extension, the lever arm was set at 75° extension, calculated on the maximum knee extension of each participant. Subjects had to push as much as possible, with both legs, on the shin pad for 5 s. Differently, during maximal isokinetic bilateral knee extension, flexion participants pushed and pulled the shin pad as fast as possible for five times uninterruptedly. The velocity of isokinetic movement was set at 90°/s. When testing the maximal isometric ankle plantar and dorsal flexion, the lever arm was set at 30° of plantar flexion, calculated from the maximum ankle dorsal flexion (0°) of each participant, and the foot was fixed on a support with two stripes. Subjects had to push down and pull up the ankle support as much as possible for 5 s, during extension and flexion trials. Finally, during maximal isokinetic ankle plantar flexion, extension participants had to push down and pull up the ankle support as fast as possible for five times continuously. The velocity of this isokinetic movement was set at 90°/s. All data were acquired at 1000 Hz, and analyzed as absolute strength, and relative strength (absolute strength/body weight).

### 2.3. Statistical Analysis

Difference at baseline between groups in age and BMI were tested with Kruskal–Wallis. Eta squared (η^2^) effect size statistics was computed according to: η^2^ = (H − k + 1)/(n − k), where H is the Kruskal–Wallis statistics, k is the number of groups, and n is the total number of observations [27]. For pairwise comparisons, the Dunn test was used with Bonferroni correction.

A two-way split-plot ANOVA was used to analyze the differences in relative and absolute strength, with group membership as a factor between (four levels) and type of strength measure as a factor within (7 levels). To follow-up, significant interaction effects, one-way ANOVA, or repeated measure ANOVA were used looking for the factor between or within, respectively. A *t*-test was used for pairwise comparisons and Bonferroni correction was applied for multiple comparisons.

The presence of univariate outliers was evaluated using a boxplot technique. Extreme outliers were defined as an individual score that exceed the threshold Q1-3.0xIQR or Q3+3.0xIQR, and moderate ones with the threshold of Q1-1.5xIQR or Q3+1.5xIQR. Normal distribution of the data was assessed via a Shapiro–Wilk test and Q–Q plots. To test the assumption of the ANOVA, the Box test was used to check the equality of a co-variance matrix with an α = 0.01. Sphericity assumption was tested with a Mauchly test and, if it is not met, Greenhouse–Geisser correction was applied. Levene’s test was used to check the assumption of equality of variances.

Results were expressed as mean and standard deviation, if they are normally distributed, or median (IQR) otherwise. Statistical analysis was performed with RStudio (RStudio Team (2020). RStudio: Integrated Development Environment for R. RStudio, PBC, Boston, MA URL http://www.rstudio.com/).

## 3. Results

### 3.1. Sociodemographic Characteristics and Baseline Comparisons

One-hundred and forty-one elderly were recruited. The LTR consisted of 15 individuals, the KTR of 46, the OB of 0, and HEG of 30. Sociodemographic characteristics were reported in Table 1. The outliers’ analysis revealed that there were not extreme outliers for age or BMI in any group. However, 28 moderate outliers were found in the OB, and 7 in the HEG for the variable age. In a similar manner, BMI showed 14 moderate outliers in the HEG, and 7 in the OB. Due to the large amount of individuals detected and the absence of absolute exclusion criteria for univariate outliers, researchers chose not to exclude the participants.

Baseline comparisons were conducted via a Kruskal–Wallis test as the variables were not normally distributed. Differences in age and BMI were examined according to group membership. Baseline demographics (age, heigth, weigth and BMI) were tested splitting the entire sample according to the factor sex (males and females). Age did not differ between groups in men or women. One way ANOVA revealed significant differences between different group of male participants in height, F (3, 80) = 3.209, *p* = 0.0274, weight F (3, 80) = 39.84, *p* < 0.0001 and BMI F (3, 80) = 61.19, *p* < 0.001. Male HEG are significantly higher (*p* = 0.0188) than male KTR. For the other variables, only OB significantly differ from all other groups (*p* < 0.0001 for all). If not mentioned, the comparisons were not significant. The Kruskal–Wallis test highlighted a significant difference in female participants between groups on the variable height (H = 88.014, df = 2, *p* = 0.01227). Wilcoxon pairwise comparisons revealed that HEG are higher than OB (*p* = 0.011). The results of a one-way ANOVA with group as between factor showed differences in women on weight, F (2,53) = 43.89, *p* < 0.0001, and BMI, F (2,53) = 80.64, *p* < 0.0001. However, only OB differs significantly from all the other groups (all *p* < 0.0001).

### 3.2. Absolute Strength

**Figure 1:**
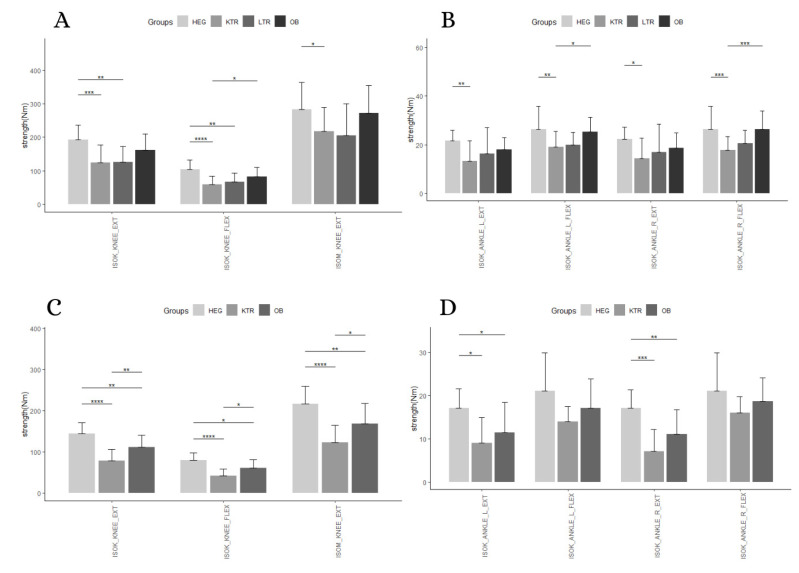
Pairwise comparison between group levels for absolute strength. (**A**): knee strength for men; (**B**): ankle strength for men; (**C**): knee strength for women; (**D**): ankle strength for women. Abbreviation: *: *p* < 0.05; **: *p* < 0.01; ***: *p* < 0.001; ****: *p* < 0.0001; HEG: healthy group; KTR: kidney transplant recipient group; LTR: liver transplant recipient group; OB: elderly with obesity group; ISOK: isokinetic muscular strength; ISOM: isometric muscular strength; EXT: extension; FLEX: flexion; R: right; L: left.

A statistically significant interaction between measurement and group membership was found for absolute strength in men (*p* = 0.006), and women (*p* < 0.0001). Simple main effects for group were significant at each measurement for men, with *p* < 0.05; in women, the strength measure of the knee and isokinetic strength of right ankle in extension showed significant simple main effects (*p* < 0.001). A significant simple main effect for measurement was found for men (*p* < 0.0001) and women (*p* < 0.0001) (Table 2 and Table 3, Figure 1).

Following up on the main effect, pairwise comparisons showed that HEG men and women performed better in all the strength measures with respect to KTR, with almost all the *p* < 0.05. However, HEG were stronger than LTR (*p* < 0.05) only in isokinetic strength of the knee’s flexors and extensor muscles in men. Finally, men showed no significant differences between HEG and OB (*p* < 0.05), while, in women, OB performed better on ankle extensor strength, knee extensors, flexor strength, and isometric strength. Comparing the pathological male elderly, muscle strength of LTR did not differ from KTR and OB groups, while muscle flexors of knees and both ankles were significantly higher in OB compared to LTR (*p* < 0.05) (Table 4). Finally, in females, muscle strength of knees was significantly higher in OB compared with KTR (*p* < 0.05), while no differences were found for ankle strength (Table 5). Mean and standard deviations of absolute muscular strength was extensively reported as Supplementary Materials (Table S1).

### 3.3. Relative Strength

A statistically significant interaction between measurement and group membership was found for relative strength in men (*p* = 0.0001), and women (*p* < 0.0001). Simple main effects for groups were significant at each measurement for men (*p* < 0.05) and women (*p* < 0.001). A significant simple main effect for measurement was found for men (*p* < 0.0001), and women (*p* < 0.0001) (Table 6 and Table 7).

**Figure 2:**
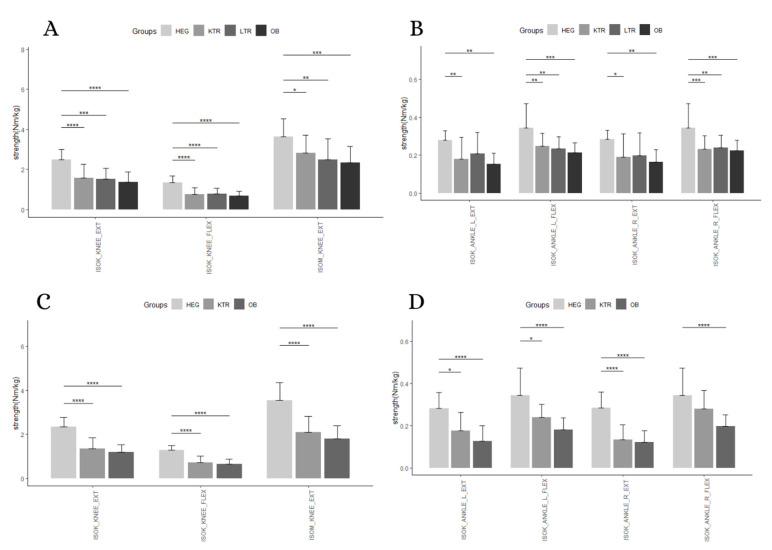
Pairwise comparison between group levels for relative strength. (**A)**: knee strength for men; (**B)**: ankle strength for men; (**C)**: knee strength for women; (**D)**: ankle strength for women. Abbreviation: *: *p* < 0.05; **: *p* < 0.01; ***: *p* < 0.001; ****: *p* < 0.0001; HEG: healthy group; KTR: kidney transplant recipient group; LTR: liver transplant recipient group; OB: elderly with obesity group; ISOK: isokinetic muscular strength; ISOM: isometric muscular strength; EXT: extension; FLEX: flexion; R: right; L: left.

Following up on the main effect, pairwise comparisons showed that HEG men and women performed better in all the relative strength measure with respect to KTR (*p* < 0.005), except for flexors of right ankles in women. However, HEG men were stronger than LTR (*p* < 0.05) in all parameters, except for ankle extensors. Finally, HEG performed better than OB in all strength parameters (*p* < 0.05), in both genders. Finally, no differences were found between pathological groups (Table 4 and Table 5, Figure 2). Mean and standard deviations of relative muscular strength were extensively reported as Supplementary Materials (Table S1).

## 4. Discussion

The aim of the present study was to understand if non-communicable disease affected the lower limb strength profile of pathological older adults with respect to their healthy counterparts. Our main findings are that healthy older adults are significantly stronger than OB, KTR, and LTR patients in absolute and relative muscular strength in all the measures gathered. Moreover, KTR presented the lower level of absolute muscular strength, while the worse performances of relative muscular strength are for the elderly with obesity.

The elderly with kidney transplants showed a significantly lower level of muscular strength than their healthy peers. This result is in agreement with the current literature; in fact, low muscle strength is common in kidney transplant recipients [28]. Even if patients living with a kidney transplant showed improvement in quality of life and survival rates compared to those who are dialysis-dependent, and the prevalence of frailty among these patients seems to remain [29]. Indeed, in KTRs, the prevalence of sarcopenia varied according to the diagnostic criteria but low muscle mass, low muscle function, and low physical performance are relatively common conditions [30].

Isokinetic muscular strength of the elderly with liver transplants is lower than HEG. However, only isokinetic knee extension and flexion differ significantly. Similarly to KTRs, patients attending liver transplantations are characterized by frailty, which persists after the transplantation [31]. However, in the first two years after liver transplantation, quality of life tends to increase rapidly, and remains stable after the achievement of almost normal values [32]. Compared to kidney transplantation guidelines, Italian guidelines for liver transplantation discourage transplantation in subjects with more than 65 years old due less liver availability [33]. In our study, we hypothesized that patients with liver transplantation were more than 62 years old, suggesting that functional evaluations were performed years after surgery. For these reasons, we speculated that the return to a “normal” life may determine the recovery of lower muscular strength, without a significant difference in the healthy elderly. However, no difference was found between the time of the evaluation from the transplantation (liver vs. kidney), so future investigations are necessary to evaluate the modification of muscular strength after liver transplantation.

People affected by obesity are generally reported to perform better in terms of absolute strength at all ages [34] with respect to the general population; however, this advantage is lost when muscular strength is considered in relation to body weight. The reason for a greater absolute strength lies in the chronic overload of the antigravity muscles, which should act as a stimulus to increase muscular strength and hypertrophy [35]. In contrast to previous studies [36,37], the absolute lower limb strength of the elderly with obesity results in being lower than healthy subjects, such as the relative lower limb muscular strength. Lower limb muscular strength for obese subjects is correlated with their level of physical activity [38]; in fact, sedentary obese results in being weaker than active obese due to the absence of overload stimulus on muscular apparatus [34].

### Limitations

The present work is a secondary data analysis and it has major limitations. In fact, data were not obtained with the aim of determining the strength profile of different non-communicable diseases. Moreover, the statistical design used was not balanced, and it could hamper the analysis [39]. Then, even if they are smaller, baseline differences in age are present, and they could account for part of the variability of the data, but, in more detail, they could hamper the generalizability of the results.

The lack of information about body composition could not explain in depth the strength difference of the four sub-groups. Nevertheless, several studies showed that muscle strength is more important than muscle mass to determine functional impairment and poor health in the elderly [40,41,42].

Finally, the absence of data about the habitual physical activity performed during the week prevents us from better explaining the reason for strength differences.

## 5. Conclusions

The results of this paper showed that the strength profile of elderly subjects is affected by non-communicable chronic disease. In more detail, elderly patients living with kidney transplants showed the lower level of absolute muscular strength, while relative muscular strength is mainly lacking in the elderly with obesity. Contrary to the authors’ hypothesis, absolute strength of participants affected by obesity was lower than healthy participants. However, no differences were found among knee and ankle joints. These results underlie the need to study in depth which type of muscle contraction could be evaluated in different chronic diseases, especially in relation to daily activities and quality of life. Future research, with more balanced samples, could take into account muscular strength changes in elderly subjects and implement guidelines for exercise prescription for the elderly with non-communicable chronic diseases.

## Figures and Tables

**Table 1:** Socio-demographics characteristics.

Measures	Sex	KTR (46)	LTR (15)	OB (50)	HEG (30)
Number	M	36	14	18	16
	F	10	1	32	14
Age (y)	M	66.64 ± 4.92	66 ± 4.39	65.28 ± 3.49	64.31 ± 4.13
	F	65.9 ± 3.87		63.41 ± 4.22	63.79 ± 5.47
Height (m)	M	1.74 ± 0.06	1.7 ± 0.08	1.72 ± 0.05	1.77 ± 0.04
	F	1.6 ± 0.06		1.57 ± 0.06	1.63 ± 0.06
Weight (kg)	M	77.44 ± 11.99	82.26 ± 14.91	120.19 ± 18.53	78.25 ± 13.49
	F	60.09 ± 10.93		96.05 ± 14.8	62.14 ± 12.88
BMI (kg/m2)	M	25.63 ± 3.12	28.32 ± 4.07	40.41 ± 5.52	24.97 ± 4.01
	F	23.44 ± 3.54		39.55 ± 5.27	23.31 ± 3.98
MMSE	M	28.42 ± 1.58	29 ± 1.41	29.6 ± 0.74	29.69 ± 0.48
	F	28.41 ± 1.87		29.38 ± 0.74	29.5 ± 0.52
Time transplant (months)	M	34.7 ± 63.61	28.43 ± 43.5		
	F	5.75 ± 7.09			
Hypertension	M	24	2	10	2
	F	5		17	1
DMT2	M	8	5	7	1
	F	0		10	0
Dyslipidemia	M	8	1	4	0
	F	3		10	1

Abbreviation: HEG: healthy group; KTR: kidney transplant recipient group; LTR: liver transplant recipient group; OB: elderly with obesity group; BMI: body mass index; SD; standard deviation; MMSE: mini mental state examination; DMT2: Type 2 Diabetes mellitus.

**Table 2:** Split plot ANOVA and post hoc tests results for absolute lower limb muscular strength of men.

Effect	Variables	dof, dofE	F	*p*	Partial η2 (90%CI)
Measure x Group	Absolute Strength	4.47, 105.88	3.61	0.006	0.13 (0.02–0.2)
Measure		1.49, 105.88	563.1	<0.0001	0.89 (0.85–0.91)
Group		3.00, 71.00	7.31	0.0002	0.24 (0.08–0.34)
Post Hoc	Variables	dof, dofE	F	*p*	Partial η2 (90%CI)
For group	ISOK_ANKLE-L_EXT	3.00, 75	4.66	0.005	0.16 (0.03–0.26)
	ISOK_ANKLE-R_EXT	3.00, 73	3.63	0.017	0.13 (0.01–0.23)
	ISOK_KNEE_EXT	3.00, 74	8.25	0.0001	0.25 (0.1–0.36)
	ISOK_ANKLE-L_FLEX	3.00, 75	5.90	0.001	0.19 (0.05–0.29)
	ISOK_ANKLE-R_FLEX	3.00, 73	8.45	0.0001	0.26 (0.1–0.36)
	ISOK_KNEE_FLEX	3.00, 74	10.85	<0.0001	0.31 (0.15–0.41)
	ISOM_KNEE_EXT	3.00, 80	4.38	0.007	0.14 (0.02–0.24)
For measure	HEG	6.00, 90.00	176.99	<0.0001	0.92 (0.89–0.93)
	KTR	1.44, 43.09	210.65	<0.0001	0.88 (0.81–0.91)
	LTR	1.31, 14.37	75.9	<0.0001	0.87 (0.71–0.91)
	OB	1.73, 25.88	154.73	<0.0001	0.91 (0.84–0.94)

**Table 3:** Split plot ANOVA and post hoc tests results for absolute lower limb muscular strength of women.

Effect	Variables	dof, dofE	F	*p*	Partial η2 (90%CI)
Measure x Group	Absolute Strength	3.44, 75.62	8.21	<0.0001	0.27 (0.11–0.37)
Measure		1.72, 75,62	489.75	<0.0001	0.92 (0.89–0.93)
Group		2, 44	14.05	<0.0001	0.40 (0.18–0.52)
Post Hoc	Variables	dof, dofE	F	*p*	Partial η2 (90%CI)
For group	ISOK_ANKLE-L_EXT	2.00, 45	5.41	0.008	0.19 (0.03–0.33)
	ISOK_ANKLE-R_EXT	2.00, 45	11.04	0.0001	0.33 (0.13–0.46)
	ISOK_KNEE_EXT	2.00, 47	15.92	<0.0001	0.40 (0.21–0.53)
	ISOK_ANKLE-L_FLEX	2.00, 45	2.76	0.074	0.11 (0–0.24)
	ISOK_ANKLE-R_FLEX	2.00, 45	1.59	0.215	0.07 (0–0.18)
	ISOK_KNEE_FLEX	2.00, 47	11.59	0.0001	0.33 (0.14–0.46)
	ISOM_KNEE_EXT	2.00, 53	11.75	0.0001	0.31 (0.13–0.43)
For measure	HEG	6.00, 78.00	270.34	<0.0001	0.95 (0.93–0.96)
	KTR	1.53, 10.72	106.72	<0.0001	0.94 (0.82–0.96)
	OB	1.83, 43.92	291.38	<0.0001	0.92 (0.88–0.94)

Abbreviations: HEG: healthy group; KTR: kidney transplant recipient group; LTR: liver transplant recipient group; OB: elderly with obesity group; dof: degree of freedom; dofE: Error degree of freedom; CI: confidence interval; ISOK: isokinetic muscular strength; ISOM: isometric muscular strength; EXT: extension; FLEX: flexion; R: right; L: left.

**Table 4:** Pairwise comparison between group levels for absolute and relative strength in men.

Outcomes	Group 1	Group 2	n1	n2	Absolute p.adj	Relative p.adj
ISOK_ANKLE-L_EXT	HEG	KTR	16	36	0.0032	0.005
	HEG	LTR	16	14	0.402	0.387
	HEG	OB	16	18	1	0.0018
	LTR	KTR	14	36	1	1
	LTR	OB	14	18	1	0.809
	KTR	OB	36	18	0.256	1
ISOK_ANKLE-R_EXT	HEG	KTR	16	36	0.012	0.0176
	HEG	LTR	16	14	0.485	0.157
	HEG	OB	16	18	1	0.0058
	LTR	KTR	14	36	1	1
	LTR	OB	14	18	1	1
	KTR	OB	36	18	0.496	1
ISOK_KNEE_EXT	HEG	KTR	16	36	0.0001	<0.0001
	HEG	LTR	16	14	0.0033	0.0003
	HEG	OB	16	18	0.42	<0.0001
	LTR	KTR	14	36	1	1
	LTR	OB	14	18	0.349	1
	KTR	OB	36	18	0.0778	1
ISOK_ANKLE-L_FLEX	HEG	KTR	16	36	0.0049	0.001
	HEG	LTR	16	14	0.1	0.0041
	HEG	OB	16	18	1	0.0001
	LTR	KTR	14	36	1	1
	LTR	OB	14	18	0.238	1
	KTR	OB	36	18	0.0177	1
ISOK_ANKLE-R_FLEX	HEG	KTR	16	36	0.0008	0.0002
	HEG	LTR	16	14	0.176	0.009
	HEG	OB	16	18	1	0.0005
	LTR	KTR	14	36	1	1
	LTR	OB	14	18	0.166	1
	KTR	OB	36	18	0.0007	1
ISOK_KNEE_FLEX	HEG	KTR	16	36	<0.0001	<0.0001
	HEG	LTR	16	14	0.0021	<0.0001
	HEG	OB	16	18	0.146	<0.0001
	LTR	KTR	14	36	1	1
	LTR	OB	14	18	0.643	1
	KTR	OB	36	18	0.0291	1
ISOM_KNEE	HEG	KTR	16	36	0.0488	0.0256
	HEG	LTR	16	14	0.0578	0.0059
	HEG	OB	16	18	1	0.0004
	LTR	KTR	14	36	1	1
	LTR	OB	14	18	0.119	1
	KTR	OB	36	18	0.113	0.356

**Table 5:** Pairwise comparison between group levels for absolute and relative strength in women.

Outcomes	Group 1	Group 2	n1	n2	Absolute p.adj	Relative p.adj
ISOK_ANKLE-L_EXT	HEG	KTR	14	10	0.0163	0.0135
	HEG	OB	14	32	0.0265	<0.0001
	KTR	OB	10	32	1	0.401
ISOK_ANKLE-R_EXT	HEG	KTR	14	10	0.0002	<0.0001
	HEG	OB	14	32	0.0029	<0.0001
	KTR	OB	10	32	0.173	1
ISOK_KNEE_EXT	HEG	KTR	14	10	<0.0001	<0.0001
	HEG	OB	14	32	0.003	<0.0001
	KTR	OB	10	32	0.0096	0.886
ISOK_ANKLE-L_FLEX	HEG	KTR	14	10	0.087	0.0257
	HEG	OB	14	32	0.308	<0.0001
	KTR	OB	10	32	0.823	0.262
ISOK_ANKLE-R_FLEX	HEG	KTR	14	10	0.254	0.306
	HEG	OB	14	32	0.835	<0.0001
	KTR	OB	10	32	0.92	0.0813
ISOK_KNEE_FLEX	HEG	KTR	14	10	0.0001	<0.0001
	HEG	OB	14	32	0.0125	<0.0001
	KTR	OB	10	32	0.0346	1
ISOM_KNEE	HEG	KTR	14	10	<0.0001	<0.0001
	HEG	OB	14	32	0.0072	<0.0001
	KTR	OB	10	32	0.0312	0.625

Abbreviation: p.adj: *p*-value adjusted for Bonferroni test; HEG: HEG group; KTR: kidney transplant recipient group; LTR: liver transplant recipient group; OB: elderly with obesity group; ISOK: isokinetic muscular strength; ISOM: isometric muscular strength; EXT: extension; FLEX: flexion; R: right; L: left.

**Table 6:** Split plot ANOVA and post hoc tests results for relative lower limb muscular strength of men.

Effect	Variables	dof, dofE	F	*p*	Partial η2 (90%CI)
Measure x Group	Absolute Strength	4.7, 104.23	5.88	0.0001	0.20 (0.08–0.28)
Measure		1.49, 104.23	540.24	<0.0001	0.89 (0.85–0.91)
Group		3.00, 70.00	11.2	<0.0001	0.32 (0.16–0.43)
Post Hoc	Variables	dof, dofE	F	*p*	Partial η2 (90%CI)
For group	ISOK_ANKLE-L_EXT	3.00, 73	5.65	0.002	0.19 (0.05–0.29)
	ISOK_ANKLE-R_EXT	3.00, 73	4.56	0.006	0.16 (0.030–0.25)
	ISOK_KNEE_EXT	3.00, 74	12.01	<0.0001	0.33 (0.17–0.43)
	ISOK_ANKLE-L_FLEX	3.00, 75	8.22	0.0001	0.25 (0.097–0.35)
	ISOK_ANKLE-R_FLEX	3.00, 73	8.02	0.0001	0.25 (0.09–0.35)
	ISOK_KNEE_FLEX	3.00, 74	16.77	<0.0001	0.41 (0.24–0.50)
	ISOM_KNEE_EXT	3.00, 80	6.63	0.0005	0.20 (0.06–0.30)
For measure	HEG	6.00, 90.00	227.606	<0.001	0.94 (0.91–0.95)
	KTR	1.45, 45.1	225.901	<0.001	0.88 (0.64–0.82)
	LTR	1.32, 13.21	94.443	<0.001	0.90 (0.72–0.92)
	OB	1.45, 24.59	120.18	<0.001	0.87 (0.75–0.91)

**Table 7:** Split plot ANOVA and post hoc tests results for relative lower limb muscular strength of women.

Effect	Variables	dof, dofE	F	*p*	Partial η2 (90%CI)
Measure x Group	Absolute Strength	2.92, 59.79	24.87	<0.0001	0.55 (0.38–0.63)
Measure		1.46, 59.79	477.82	<0.0001	0.92 (0.89–0.94)
Group		2, 41	45.17	<0.0001	0.69 (0.53–0.76)
Post Hoc	Variables	dof, dofE	F	*p*	Partial η2 (90%CI)
For group	ISOK_ANKLE-L_EXT	2.00, 43	18.72	<0.0001	0.47 (0.26–0.58)
	ISOK_ANKLE-R_EXT	2.00, 43	30.43	<0.0001	0.59 (0.4–0.68)
	ISOK_KNEE_EXT	2.00, 47	40.92	<0.0001	0.64 (0.47–0.72)
	ISOK_ANKLE-L_FLEX	2.00, 45	16.86	<0.0001	0.43 (0.23–0.55)
	ISOK_ANKLE-R_FLEX	2.00, 45	12.98	<0.0001	0.37 (0.16–0.49)
	ISOK_KNEE_FLEX	2.00, 47	33.67	<0.0001	0.59 (0.41–0.68)
	ISOM_KNEE_EXT	2.00, 52	33.48	<0.0001	0.56 (0.39–0.65)
For measure	HEG	6.00, 78.00	238.24	<0.0001	0.95 (0.93–0.96)
	KTR	1.56, 9.36	83.36	<0.0001	0.94 (0.79–0.95)
	OB	1.58, 34.83	237.78	<0.0001	0.92 (0.86–0.94)

Abbreviations: HEG: healthy group; KTR: kidney transplant recipient group; LTR: liver transplant recipient group; OB: elderly with obesity group; dof: degree of freedom; dofE: Error degree of freedom; CI: confidence interval; ISOK: isokinetic muscular strength; ISOM: isometric muscular strength; EXT: extension; FLEX: flexion¸ R: right; L: left.

## Supplementary Materials

The following are available online at https://www.mdpi.com/2308-3417/5/4/83/s1, Table S1: Overall and between groups descriptive statistics for absolute and relative lower limb muscular strength of men and women (mean ± SD).
