# Supplementary material for: Lower Limb Strength Profile in Elderly with Different Pathologies: Comparisons with Healthy Subjects
Source: Geriatrics (Basel). 2020 Oct 22;5(4):83. doi: 10.3390/geriatrics5040083 (PMC7709664; doi:10.3390/geriatrics5040083)
Supplement: Supplementary file 1 [file geriatrics-05-00083-s001.pdf]

Table S1. Overall and between groups descriptive statistics for absolute and relative lower limb muscular strength (mean ± SD).

Outcomes	Group	Sex (num)	Absolute muscular strength (Nm)	Relative muscular strength (Nm/body weight)
ISOK_ANKLE-L_EXT	HEG	M(16)	21.58 ± 4.26	0.28 ± 0.05
		F(14)	17.07 ± 4.43	0.28 ± 0.08
	LTR	M(14)	16.17 ± 10.84	0.21 ± 0.11
		F(1)		
	KTR	M(36)	13.21 ± 8.51	0.18 ± 0.12
		F(10)	9.04 ± 5.89	0.18 ± 0.09
	OB	M(18)	17.88 ± 5.09	0.16 ± 0.06
		F(32)	11.44 ± 7.02	0.13 ± 0.07
ISOK_ANKLE-R_EXT	HEG	M(16)	22.21 ± 4.99	0.29 ± 0.05
		F(14)	17.12 ± 4.22	0.28 ± 0.08
	LTR	M(14)	16.81 ± 11.69	0.2 ± 0.12
		F(1)		
	KTR	M(36)	14.4 ± 8.35	0.19 ± 0.12
		F(10)	7.08 ± 5.03	0.13 ± 0.07
	OB	M(18)	18.69 ± 6.08	0.16 ± 0.07
		F(32)	11.12 ± 5.56	0.12 ± 0.05
ISOK_KNEE_EXT	HEG	M(16)	193.21 ± 43.57	2.49 ± 0.5
		F(14)	143.81 ± 27.34	2.35 ± 0.41
	LTR	M(14)	126.56 ± 45.32	1.53 ± 0.53
		F(1)		
	KTR	M(36)	123.84 ± 53.85	1.58 ± 0.69
		F(10)	78.67 ± 27.03	1.35 ± 0.5
	OB	M(18)	161.59 ± 48.42	1.38 ± 0.49
		F(32)	111.14 ± 28.85	1.19 ± 0.34
ISOK_ANKLE-L_FLEX	HEG	M(16)	26.31 ± 9.38	0.34 ± 0.13
		F(14)	21 ± 8.89	0.34 ± 0.13
	LTR	M(14)	19.89 ± 5.15	0.23 ± 0.06
		F(1)		
	KTR	M(36)	19.04 ± 6.44	0.25 ± 0.07
		F(10)	13.96 ± 3.47	0.24 ± 0.06
	OB	M(18)	25.31 ± 5.92	0.21 ± 0.05
		F(32)	17.11 ± 6.7	0.18 ± 0.06
ISOK_ANKLE-R_FLEX	HEG	M(16)	26.31 ± 9.38	0.34 ± 0.13
		F(14)	21 ± 8.89	0.34 ± 0.13
	LTR	M(14)	20.47 ± 5.4	0.24 ± 0.07
		F(1)		
	KTR	M(36)	17.84 ± 5.51	0.23 ± 0.07
		F(10)	16 ± 3.7	0.28 ± 0.09
	OB	M(18)	26.38 ± 7.49	0.22 ± 0.06
		F(32)	18.67 ± 5.36	0.2 ± 0.05
ISOK_KNEE_FLEX	HEG	M(16)	103.77 ± 28.29	1.34 ± 0.34
		F(14)	79.45 ± 17.4	1.29 ± 0.21
	LTR	M(14)	66.56 ± 27.14	0.79 ± 0.26
		F(1)		
	KTR	M(36)	59.38 ± 24.72	0.77 ± 0.32
		F(10)	42.2 ± 16.39	0.73 ± 0.3
	OB	M(18)	82.51 ± 27.77	0.69 ± 0.23
		F(32)	60.64 ± 20.33	0.65 ± 0.23
ISOM_KNEE	HEG	M(16)	282.77 ± 80.71	3.63 ± 0.89
		F(14)	216 ± 43.71	3.55 ± 0.8
	LTR	M(14)	205.62 ± 94.63	2.49 ± 1.05
		F(1)		

KTR	M(36)	217.95 ± 70.8	2.83 ± 0.88
	F(10)	123.3 ± 40.79	2.11 ± 0.72
OB	M(18)	272.98 ± 82.51	2.33 ± 0.84
	F(32)	168.27 ± 49.46	1.8 ± 0.59

Abbreviation: HEG: healthy group; KTR: kidney transplant recipient group; LTR: liver transplant recipient group; OB: elderly with obesity group; M: male; F: female; ISOK: isokinetic muscular strength; ISOM: isometric muscular strength; EXT: extension; FLEX: flexion; R: right; L: left; SD: standard deviation.
